# Preliminary Efficacy of Adapted Responsive Teaching for Infants at Risk of Autism Spectrum Disorder in a Community Sample

**DOI:** 10.1155/2015/386951

**Published:** 2015-01-11

**Authors:** Grace T. Baranek, Linda R. Watson, Lauren Turner-Brown, Samuel H. Field, Elizabeth R. Crais, Linn Wakeford, Lauren M. Little, J. Steven Reznick

**Affiliations:** University of North Carolina at Chapel Hill, Chapel Hill, NC 27599-7122, USA

## Abstract

This study examined the (a) feasibility of enrolling 12-month-olds at risk of ASD from a community sample into a randomized controlled trial, (b) subsequent utilization of community services, and (c) potential of a novel parent-mediated intervention to improve outcomes. The First Year Inventory was used to screen and recruit 12-month-old infants at risk of ASD to compare the effects of 6–9 months of Adapted Responsive Teaching (ART) versus referral to early intervention and monitoring (REIM). Eighteen families were followed for ~20 months. Assessments were conducted before randomization, after treatment, and at 6-month follow-up. Utilization of community services was highest for the REIM group. ART significantly outperformed REIM on parent-reported and observed measures of child receptive language with good linear model fit. Multiphase growth models had better fit for more variables, showing the greatest effects in the active treatment phase, where ART outperformed REIM on parental interactive style (less directive), child sensory responsiveness (less hyporesponsive), and adaptive behavior (increased communication and socialization). This study demonstrates the promise of a parent-mediated intervention for improving developmental outcomes for infants at risk of ASD in a community sample and highlights the utility of earlier identification for access to community services earlier than standard practice.

## 1. Introduction

The rising prevalence of autism spectrum disorder (ASD) [1] and relatively late age of diagnosis for a neurodevelopmental disorder of early onset [2, 3] highlight the need for more efficacious methods of identifying and intervening with infants at risk of a later diagnosis of ASD. Early identification and intervention can serve to optimize developmental outcomes, decrease disability, and ultimately reduce associated burdens on families and society. In the United States, federal law mandates state agencies to administer early intervention (EI) services under Part C of the Individuals with Disabilities Education Act [4]; however, state eligibility criteria vary tremendously and, in most cases, infants at risk for ASD are not eligible for these services unless they have an established condition (e.g., clinical diagnosis) and/or a significant level (e.g., >25%) of developmental delay on standardized assessments [5].

Moreover, physicians typically do not screen for ASD prior to 18–24 months of age and are reluctant to make referrals to EI without evidence of available and efficacious treatments for such young children [6–8]. Although some studies with toddlers (ages 15–36 months) fail to show significant effects of tested interventions on child language, cognition, or ASD symptoms [9–11], growing evidence shows that early behavioral interventions are beneficial for some toddlers with clear ASD symptoms [12–16]. However, intervention studies differ vastly in terms of design, content, format, intensity, time course, age at enrollment, and their outcome measures and moderating variables, which obfuscates the key ingredients responsible for positive outcomes. More research utilizing strong comparison groups would help to determine benefits of specific treatments [17], and integrating the perspectives of families and other stakeholders is needed to help translate promising interventions into community settings [18].

Five published randomized controlled trials (RCTs) specifically included toddlers with ASD < 24 months of age and thus are described further. These studies utilized clinical samples of children with ASD referred from EI programs or clinics and/or high-risk infant siblings. Although the specific approach and context of the interventions (clinic versus in-home) varied, all five studies demonstrated positive effects of early behavioral intervention [12–16]. Specifically, Dawson et al. [13] found evidence that the Early Start Denver Model (ESDM; [19]) was more effective than usual services for improving cognitive and adaptive functioning for 48 children, ages 18–30 months (*M* = 23). ESDM, a relatively high intensity intervention based on principles of applied behavior analysis and developmental strategies, was administered by trained clinicians in the families' homes ~15 hours per week and supplemented by parents over the course of two years.

Landa et al. [15] compared an intervention called interpersonal synchrony against a noninterpersonal synchrony intervention with 50 toddlers ages 20 to 33 months (*M* = 28). Both interventions were provided in a classroom 10 hours per week for 6 months and were supplemented by 38 hours of parent education and 1.5 hours per month of home-based parent training. Children in both groups showed significant gains in social, language, and cognitive outcomes across time, with children in the interpersonal synchrony intervention showing significantly larger gains in socially engaged imitation.

Carter et al. [12] compared a relatively lower intensity behavioral intervention, Hanen's More than Words (HMTW) [20], to services as usual with 62 toddlers ages 15–25 months (*M* = 20). The HMTW program comprised eight group parent training sessions and three individual in-home parent-child sessions provided by speech-language pathologists over ~5 months to teach parents practical communication strategies. Child communication outcomes were moderated by baseline levels of “object interest,” such that children with lower levels of object interest fared better with HMTW, whereas those with higher levels of object interest did better in the control condition. These findings suggest that subgroups of children may respond differentially to various types of behavioral interventions.

Kasari et al. [14] applied a wait-list control design with 38 children with ASD ages 21–36 months (*M* = 31) and demonstrated that an 8-week caregiver-mediated intervention was effective for improving some skills (i.e., joint engagement, response to joint attention, and functional play acts) but not others (i.e., initiations to joint attention and diversity in play acts). The immediate treatment group maintained skills one year after treatment. Interestingly, utilization of community EI services was not predictive of outcomes in this relatively short timeframe. Finally, an RCT by Schertz et al. [16] similarly studied the effects of a focused, developmentally sequenced intervention model called Joint Attention Mediated Learning (JAML) on the acquisition of social-communication competencies. They randomized 23 toddlers with ASD all under 30 months of age (*M* = 26 months) and found that the intervention group outperformed the usual services group on observed measures of responding to joint attention and focusing on faces. Intervention x time effects also favored the JAML group on standardized measures of receptive language and functional communication. Although both of the latter joint attention studies used a parent-mediated approach, the Kasari study was conducted in a clinical setting, whereas the JAML was conducted in the families' homes. Overall, despite differences in approach and intensity, all five of these RCTs show positive effects of early behavioral interventions relative to comparison conditions (services as usual in four cases, and an active treatment in Landa et al. [15]) for toddlers with clear symptoms of ASD from clinical populations, particularly for social-communication outcomes. However, no studies have been published that test the effects of a parent-mediated intervention for infants at risk of ASD (before diagnosis) from a nonclinical community population, which is the prime aim of the current study.

Neurodevelopmental theories stress the importance of timing and transactions between genetic susceptibilities to ASD and environmental factors that shape neural connections through experience [2, 3, 22, 23]. These theories, along with evidence that neural shaping processes become highly prevalent at the end of the first year of life [24], lend support to the hypothesis that behavioral interventions beginning as soon as risk for ASD can be detected will be efficacious [25]. Dawson [26] further theorized that prevention of ASD is plausible through new advances in detecting at-risk infants (before the full presentation of the syndrome) and implementing interventions that can alter the course of early behavioral and brain development for at risk infants.

Thus, development of novel and efficacious interventions for infants at risk of a later diagnosis of ASD is needed to test scientific hypotheses and address growing public health issues. Models that are socioculturally and ecologically congruent with the needs of families with very young infants and toddlers, particularly parent-mediated models, need to be considered. Parental responsiveness is a key variable associated with developmental gains in typically developing children as well as for those who are at risk of or who have been diagnosed with developmental disabilities including ASD [27–32]. Furthermore, approaches that are consistent with the guidelines set forth in Part C of the Individuals with Disabilities Education Act [4] (e.g., individualized, conducted in natural environments, and integrated into daily activities) are particularly important for utility in community EI settings. No RCTs have been published that demonstrate the efficacy of such parent-mediated interventions for very young infants and toddlers (<18 months) specifically at risk of a later diagnosis of ASD in a nonclinical sample.

This study aimed at (a) establishing the feasibility of enrolling 12-month-olds at risk of ASD in a community sample into an intervention study, (b) describing families' subsequent utilization of community EI services, and (c) evaluating the potential of a novel parent-mediated intervention, called “Adapted Responsive Teaching” (ART), to improve parental responsiveness as well as children's developmental outcomes. Empirical evidence clearly links early pivotal behaviors (e.g., social play, joint attention, arousal and attention, engagement, adaptability, and coping), similar to those used in ART, to later outcomes in children with and/or without ASD [33–37]. Thus, we hypothesized that parents in the ART group, as compared to a “referral to early intervention and monitoring” (REIM) group, would be more responsive and less directive during parent-child free-play interactions. We also hypothesized that ART would improve the infants' pivotal behaviors in two domains—social-communication and sensory-regulatory functions—and that these effects would be evident at both outcome assessments (immediately after treatment and at later follow-up). We also explored ASD diagnostic outcomes to generate hypotheses for future studies.

## 2. Method

**Figure 1:**
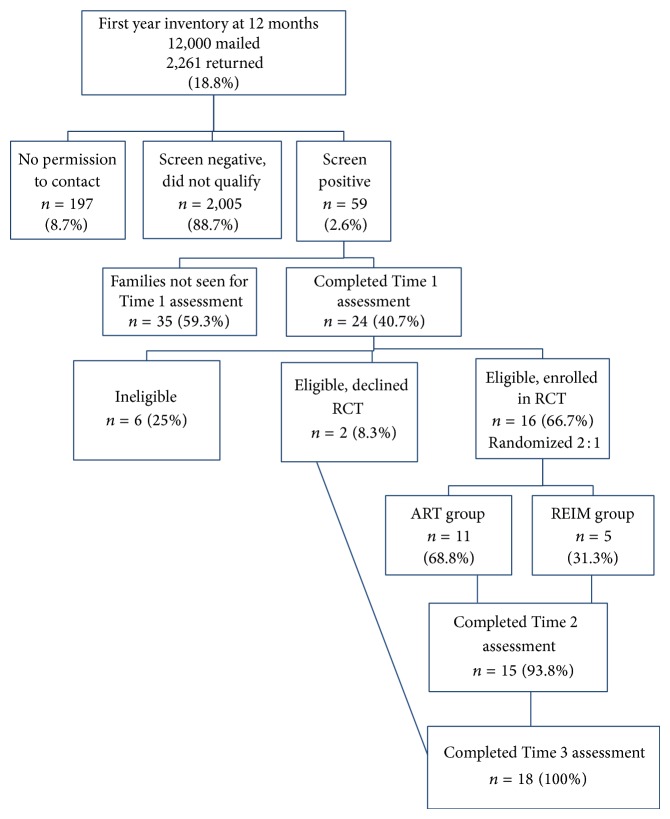
Study flow chart with participation rates for each phase.

This prospective behavioral intervention study employed a two-part procedure across ~20 months: (1) screening and ascertainment of 12-month-old infants at risk of a later diagnosis of ASD from a community sample using the First Year Inventory, version 2.0 (FYI) [38], with subsequent referral to the community EI system, and (2) enrollment of eligible parent-infant dyads into a randomized controlled trial (RCT) to investigate the preliminary efficacy of ART versus REIM and track utilization of community EI services through the duration of the study for all participants. Both ART and REIM groups received a battery of standardized assessments at each of three time points: before randomization (Time 1, around 14 months), postintervention phase (Time 2, around 22 months), and diagnostic follow-up (Time 3, around 32 months). Eligible families who declined randomization were assessed only at Time 1 and Time 3.  Figure 1 presents a flow chart detailing the study phases and participation rates at each phase. Linear and multiphase growth models were used to compare the two groups in the RCT phase on outcome variables of interest. The quantity and types of community EI services and diagnostic outcomes were analyzed descriptively for all participants.

### 2.1. Ascertainment and Recruitment of Infants at Risk of a Later Diagnosis of ASD Using FYI

Approximately 12,000 FYI screening packets were mailed to families in the catchment area on the basis of birth registry records ~2 weeks prior to the child's first birthday across a 15-month time span. The catchment area included 5 counties in central North Carolina (NC) representing both rural and urban areas. Hispanic surnames were excluded from the mailing due to the lack of availability of Spanish language FYI and ART protocols. A letter in the packet informed parents of the purpose of our study (i.e., screening for risk for autism and related developmental disorders; potential eligibility for an RCT). This recruitment strategy ensured a broader at-risk sample than one relying solely on genetically high risk younger infant siblings of children with ASD. A total of 2,261 FYIs were completed and returned by families, representing a 19% response rate. The purpose of this screening was to identify a cohort of infants from a community sample that had sufficient risk markers deemed eligible to participate in the RCT. This was not intended as an epidemiological study of screening procedures or a psychometric study of tool development; see Turner-Brown et al. [39] for details of FYI validation in a community sample, which indicated the 12-month-olds receiving at risk scores on the FYI that had a 31% chance of obtaining an ASD diagnosis by three years of age.

Families of infants who met one of the following risk criteria were invited to participate in a comprehensive developmental assessment (Time 1, before randomization) to confirm risk status: (1) infants with an FYI risk score at or above the 95th percentile, (2) infants with an FYI risk score at or above the 90th percentile with documented parent concerns in the core ASD symptom areas, and (3) any infant whose parent indicated a family history of autism spectrum disorder (ASD). Children were excluded from further study if their parents reported significant vision or hearing impairments, Down syndrome, cerebral palsy, and/or significant prematurity (<2500 grams at birth), regardless of whether or not they met FYI algorithm cut-points. A total of 59 children met preliminary inclusion criteria. Twenty-eight families agreed to participate in the Time 1 assessment, but four subsequently cancelled, yielding 24 (40.1%). Thirty-one families were either unreachable or declined the opportunity to participate further.

### 2.2. Time 1 Baseline Prerandomization Assessment

Following parental written consent, 24 infants completed a baseline developmental assessment in a family friendly assessment suite in a community adjacent to the university campus. Measures used in the assessment are explicated below. An eligibility algorithm was developed to ensure that only children with clear delays or concerns related to ASD (based on a combination of parent report and observational measures) were invited to enroll in the intervention phase of the study. This algorithm took into account all Time 1 measures (see below) and deemed eligible any infant who (1) met ASD cutoffs on diagnostic measures (i.e., AOSI ≥ 7, and/or ADOS-T ≥ 12), and/or (2) showed delays in 2/3 domains of social-communication [i.e., expressive, receptive, and social/play as measured by the MSEL (*T* < 40), CSBS (SS < 7), MCDI (<15%), and ITSEA (rated “of concern”)], and/or (3) showed disruptions in 2/3 domains of sensory-regulatory functions [i.e., sensory processing, regulatory processing, and repetitive/atypical behaviors, as measured by the SPA (>1.5 s.d. above norm for age), SEQ (>1.5 s.d. above norm for age), and ITSEA (rated “of concern”)]. Families received the results via an interpretive conference and a written report.

### 2.3. Enrollment and Randomization of Eligible Participants into the RCT Intervention Phase

Infant-parent dyads were recruited into the RCT by the project coordinator following the Time 1 assessment if they met eligibility criteria above. Of the 24 infants who completed a Time 1 assessment, six (25%) did not meet full eligibility criteria; thus, their families were not offered enrollment in this phase. The remaining 18 infants met eligibility criteria and sixteen of these families (88.9%) subsequently signed consent and were enrolled into the RCT intervention phase. All 18 also were referred to local community EI services.

Randomization of parent-infant dyads was conducted using a random number generator in Excel by an investigator blind to the assessment results. Given the small sample size and our primary aim to test the promise of a novel intervention, a 2 : 1 randomization procedure was used [40]. Thus, a total of 11 dyads were randomized into ART and 5 were randomized into the REIM condition. The project coordinator notified the families of their assignment following randomization and answered any questions. The two eligible families that declined participation in the RCT (intervention phase) agreed to complete the final diagnostic follow-up assessment (Time 3) and thus were included in the descriptive data on diagnostic outcomes. Demographics for these 18 families are provided in Table 1.

### 2.4. Assessments/Measures (All Participants)

*The First Year Inventory (FYI) [38]*. It is a 63-item parent-report screener used to identify infants at risk of ASD at 12 months. Item/scoring details are available elsewhere [41]. It has a positive predictive value of .31 [39]. The FYI was the primary screening measure for recruiting infants into the study. 

*The Mullen Scales of Early Learning (MSEL) [42]*. It is a standardized developmental assessment for birth of 58 months. It has 4 scales (Fine Motor, Visual Reception, Receptive, and Expressive Language). Two outcomes of interest (Receptive and Expressive *T* scores) were analyzed at Times 1, 2, and 3. 

*The Vineland Adaptive Behavior Scales, Second Edition (VABS-II) [43]*. It is a parent interview that assesses adaptive behaviors. The Parent/Caregiver Rating Form was used. Standard scores were computed for the three outcomes of interest: communication (breaking out receptive and expressive), and socialization at Times 1, 2, and 3. 

*The Communication and Symbolic Behavior Scale (CSBS) Developmental Profile [44]*. It is a standardized, norm-referenced instrument used to assess children with functional communication ages between 6 and 24 months. The Caregiver Questionnaire and Behavior Sample were both computed at Times 1 and 2 to examine communication and symbolic outcomes. 

*The MacArthur-Bates Communicative Development Inventory (MCDI) [45]*. It is a standardized, norm-referenced parent report of children's communication skills. The Words and Gestures form for infants aged 8–18 months was used at Time 1 for eligibility assessment. 

*The Sensory Processing Assessment (SPA) for Young Children [46]*. It is a play-based assessment that was used to measure hyperresponsiveness (i.e., approach-avoidance to sensory toys) and hyporesponsiveness (i.e., orienting responses across 3 sensory modalities). It has good interrater reliability (ICC = .91–.99), discriminates between children with ASD and those with other developmental disabilities, and shows sensitivity to maturational change [47, 48]. Summary scores for hyporesponsiveness and hyperresponsiveness were analyzed at Times 1, 2, and 3. 

*The Sensory Experiences Questionnaire (SEQ), Version 2.1 [49]*. It is a 43-item parent questionnaire for children 6 months to 6 years that measures responses to various sensory stimuli in the context of daily activities. The internal consistency is *α* = .80 [50] and it has good discriminative validity [51]. Summary scores for hyporesponsiveness and hyperresponsiveness were analyzed at Times 1, 2, and 3. 

*The Infant-Toddler Social Emotional Assessment (ITSEA) [52]*. It is a 166-item measure of social-emotional competencies/problems at 12–36 months. It was used for eligibility determinations at Time 1. 

*The Autism Observation Scale for Infants (AOSI) [53]*. It uses 18 markers to identify infants at risk of autism during semistructured play. A total score >7 was used as cut-off at Time 1 for eligibility criteria. 

*The Autism Diagnostic Observation Schedule (ADOS) [54]*. It is a standardized, semistructured assessment used to evaluate social-communication symptoms in children suspected of having ASD. We used either Module 1 or 2 at Time 3 to describe clinical outcomes in this study. 

*A Parent Diagnostic Interview*. It was conducted by the Clinical Psychologist (LTB) with all participants at Time 3. Based upon DSM-IV-TR criteria for autistic disorder, this interview covered early developmental history and diagnostic symptoms in core autism domains. This interview was used to provide parent input to support the clinical diagnosis (if any) assigned by the psychologist. 

*The Maternal Behavior Rating Scale (MBRS) [55]*. It is a 12-item (5 point) rating assessing parent interactive style across 4 factors (or dimensions): responsive/child oriented (sensitivity, responsivity, and effectiveness; 3–15 points), affect/animation (acceptance, enjoyment, expressiveness, inventiveness, warmth; 5–25 points), achievement oriented (achievement, verbal praise; 2–10 points), and directive (directiveness, pace; 2–10 pts). It is sensitive to treatment change [30]. A 10-minute parent-child free-play session with standard toys was videotaped at Times 1, 2, and 3. Ratings were scored from video by a trained coder, blind to group assignment. A second coder scored independently; consensus scores were computed for final analyses for two outcomes—responsive and directive. 

*A Demographic Form.* It was used to document information about the infant/family (e.g., race, ethnicity, gender, education, number/ages of people in the home, and child care arrangements). 

*A Monthly Status Check and Community Early Intervention Services Interview (MSC)*. It was conducted each month by the project coordinator with all families. This 15–20 minute session aimed to (a) obtain updates on the infant's general health and developmental status (e.g., doctor visits, medications, and behavior changes), (b) track referrals to and utilization of community EI services (e.g., type, dosage, provider, location, parent trainings, and provider referrals/recommendations and changes in services since previous interview), (c) answer any questions, and (d) provide general support to facilitate community referrals.

### 2.5. Additional Measures Used with ART Group Only

*The Family Routines Exploration and Description Revised (FRED) [56]*. It was developed to facilitate intervention planning. This semistructured parent interview examined the child's current level of participation in daily family routines, strategies parents use to support their child in those routines, and parents' satisfaction with their child's participation in those routines. 

*The ART Interventionist Implementation Fidelity Checklist (IFC)*. It is adapted from Mahoney and MacDonald [57] and is a 24-item checklist used to measure the extent to which interventionists appropriately engaged parents in ART. Monthly videos with each parent-infant dyad were scored for consensus by two raters. Fidelity was monitored by reviewing videos of sessions once per month for each child; fidelity scores ranged from 80 to 90% across sessions. 

*The Parent Adherence Rating Form (PARF)*. It was developed as a 10-item rating of the interventionist's perceptions of parent adherence. It was completed following each intervention session. A percent score was computed and tracked weekly. Strategies and/or pivotal behaviors were reviewed if adherence dropped below 80%. Parent adherence scores were 73–97% across sessions. 

*The Parent Weekly Log (PWL)*. It was used to track parent's self-reported ratings of understanding, ease, and success of strategy use on a weekly basis, and when to review specific strategies.

### 2.6. Intervention Phase Protocols

#### 2.6.1. General Early Intervention Referral Protocol

Following the Time 1 assessment, all infants with established risk factors were referred to the EI program in their county. Following consent and randomization, all services received by families participating in the study were systematically documented (MSC) by the project coordinator who answered general developmental questions and helped families attain access to community resources. This design did not require parents in either group to seek community EI services and did not guarantee that the family would be able to access community EI services under Part C of IDEA.

#### 2.6.2. Referral to Early Intervention and Monitoring (REIM) Group

The REIM group received EI services normally available in the local communities, depending on parent choices, resources, and infant eligibility for publicly funded EI services. They also received monthly phone calls from our project coordinator to answer questions and track their community services as noted above. They received identical assessments on the same schedule (Times 1, 2, and 3) matched to the ART group.

#### 2.6.3. Adapted Responsive Teaching Group

ART is a 6-month relationship-focused, home based intervention aimed at improving parental responsiveness and child developmental outcomes [58] that is based on the responsive teaching curriculum [30, 57]. Research indicates that parents can be taught responsive strategies (e.g., follow child's lead; imitate child, or take one turn and wait) [27] and that relationship-focused programs, particularly ≥6 months, have been associated with positive child outcomes [29, 30, 59–61]. ART employed modeling and coaching to encourage parents to use responsive strategies during daily routines with their children, which were designed to target “pivotal” behaviors (e.g., social play, joint attention, arousal and attention, engagement, adaptability, and coping) shown to benefit later development (e.g., [33–37, 62]).

**Figure 2:**
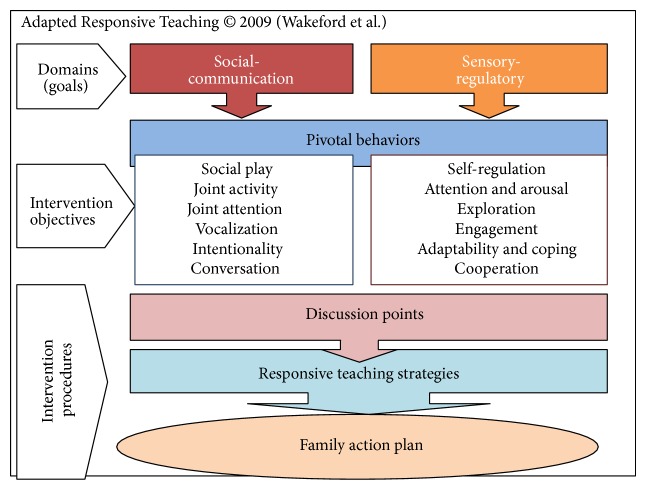
Adapted Responsive Teaching (ART) conceptual model.

ART was administered in this preliminary study with the 4 main intervention components depicted in Figure 2.* Pivotal Behavior Intervention Objectives* targeted key child developmental behaviors within the two key domains (i.e., social-communication and sensory-regulatory Functions).* Discussion points* consisted of 135 scripted miniconversations used by interventionists to discuss the rationale of ART with parents (1 or 2 selected per session).* Responsive Teaching Strategies* were taught to parents to use during daily routines to interact with their infants across 5 dimensions (i.e.,* reciprocity*,* contingency*,* control*,* affect, *and* match*.).* Family Action Plans* were “follow-through” plans for parents to reflect on, discuss with others, and/or integrate newly learned strategies into interactions with their infants between sessions. ART was adapted from responsive teaching [57] by our team to enhance its appropriateness for one-year-olds at risk of ASD. Specifically, the ART model was redesigned with two domains to match the FYI structure. Pivotal behaviors were streamlined to address a smaller subset of behaviors often observed in infants at risk of ASD. Both content and process were enhanced by adding parent education sessions using adult learning strategies. Also, supporting materials were developed (e.g., FRED; intervention notebook; weekly documentation forms).

Interventionists were three staff members with experience in child development/EI (e.g., therapists, teachers). Session plans were printed for parents weekly. Interventionists documented children's progress and parents' adherence ratings. An intervention coordinator led biweekly meetings with the intervention team and monitored progress.

The target intervention dosage was 36 contacts: 2 in-home sessions per week for the first 6 weeks = 12; 1 home session plus 1 phone call/email contact per week for second 6 weeks = 12; 1 in-home session per week for the last 12 weeks = 12. Thus, intensity was highest in the beginning and was slowly reduced as parents mastered the skills. Interventionists could flexibly increase the number of in-home sessions up to 36 (or 8 mos. maximum) to support higher implementation fidelity. Families in the ART group received an average of 33.5 (range 20–39) total contacts (in-home + phone/email) across a 6- to 8-month period. The average number of in-home sessions was 27.6 (range 18–33), and the average number of phone/email contacts was 5.8 (range 2–10).

### 2.7. Time 2 after Intervention (ART/REIM) and Time 3 Diagnostic Follow-Up Assessments

After concluding the intervention phase (ART or REIM), the toddlers (ages 22–26 months; *M* = 23.6) were scheduled for Time 2 assessments with a multidisciplinary team to measure developmental outcomes (i.e., MSEL, VABS, SEQ, SPA, CSBS, and MBRS). The diagnostic follow-up (Time 3) assessments were conducted ~6 months later (ages 30–35 months; *M* = 32.3) for all toddlers in the RCT as well as the two from the Eligible/Declined group. Time 3 included developmental outcomes but also added the blinded diagnostic evaluation by a clinical psychologist (ADOS + diagnostic interview). The assessment team was blinded to group assignment; parents were instructed to not share information regarding EI services or group assignment. An interpretive conference and a written report were provided to all families. Parents received a $20 gas card per assessment for travel costs. No families dropped out of the RCT; however, one family in the REIM group missed the Time 2 assessment. All 16 families who were enrolled in the RCT completed their Time 3 assessments as did the 2 families from the Eligible/Declined group.

### 2.8. Statistical Analyses of Treatment Effects

Initial estimates for the unknown parameters from both the linear and multiphase growth models were obtained using maximum likelihood estimation (MLE). The linear model specified that *Y*_*it*_ equals an outcome for child *i* at time *t*, and *T*_*it*_ equals the observed time (in months) from the date of the first assessment. We fit the following hierarchical linear growth model: *Y*_*it*_ = *β*_0*i*_ + *β*_1*i*_ *T*_*it*_ + *ε*_*it*_, where *ε*_*it*_ ~ *N*(0, *δ* ^2^). The level two (subject) equations were *β*_0*i*_ = *γ*_00_ + *μ*_0*i*_ for the intercept and *β*_1*i*_ = *γ*_10_ + *γ*_11_ *D*_*i*_ + *μ*_1*i*_ for the slope; *μ*_0*i*_ and *μ*_1*i*_ were assumed to follow a multivariate normal distribution. Thus,

(1) $$\begin{array}{c} \left[\mu_{0i}{,\mu }_{1i}\right]\sim MVN\left(\left[0,0\right],\left[\begin{array}{cc} \tau_{00}^{2} & \tau_{01}\\ \tau_{10} & \tau_{11}^{2} \end{array}\right]\right). \end{array}$$

For each outcome examined, we also estimated a set of models that relaxed the assumption of strictly linear growth over the period observed. Multiphase mixed effects models for repeated measure data assume that growth over time exhibits distinctively different rates of change from one time period to another [63] and requires dividing time into discrete segments or phases. In the current study, growth after the initial assessment until the cessation of intervention (Times 1 to 2) constituted the first phase, that is, active treatment, while the subsequent, postintervention follow-up period (Time 2 to 3) constituted the second phase. Similar to the linear model, the multiphase growth model specified that *Y*_*it*_ equals an outcome for child *i* at time *t*; however, the time variable only took on discreet values, *φ*_*it*_ ∈ {1,2, 3}, representing the baseline, posttreatment, and follow-up periods, respectively. We defined two within subject-level regressors:

(2) $$\begin{array}{c} \theta_{1it}=\left\{\begin{array}{cc} 0 & \text{if}\;\varphi_{it}\leq 1\\ 1 & \text{if}\;\varphi_{it}>1, \end{array}\right.\;\;\theta_{2it}=\left\{\begin{array}{cc} 0 & \text{if}\;\varphi_{it}\leq 2\\ 1 & \text{if}\;\varphi_{it}>2. \end{array}\right. \end{array}$$

The outcome was assumed to be sampled from the following model: *Y*_*it*_ = *β*_0*i*_ + *β*_1*i*_ *θ*_1*it*_ + *β*_2*i*_ *θ*_2*it*_ + *ε*_*it*_, where *ε*_*it*_ ~ *N*(0, *δ* ^2^). The level two (subject) equations were modified slightly from the linear growth model. Because growth was now captured in two parameters, random subject level variation in growth was not fully identified. We therefore only estimated fixed variation in two growth parameters as a function of treatment assignment, *β*_1*i*_ = *γ*_10_ + *γ*_11_ *D*_*i*_, and *β*_2*i*_ = *γ*_20_ + *γ*_21_ *D*_*i*_. The intercept equation still contained a random component, but in order to detect potential heteroscedasticity in the residuals induced by random, subject level differences in linear growth* within segments*, we included another random effect in an interaction with time as it was originally coded; *β*_0*i*_ = *γ*_00_ + *μ*_0*i*_ + *μ*_1*i*_ *T*_*it*_. Again, *μ*_0*i*_ and *μ*_1*i*_ were assumed to follow a multivariate normal distribution:

(3) $$\begin{array}{c} \left[\mu_{0i}{,\mu }_{1i}\right]\sim MVN\left[0,0\right],\left[\begin{array}{cc} \tau_{00}^{2} & \tau_{01}\\ \tau_{10} & \tau_{11}^{2} \end{array}\right]. \end{array}$$

## 3. Results

### 3.1. Screening and Feasibility-to-Enroll Phase

The first aim was to examine feasibility of enrolling one-year-olds at risk of ASD into an intervention study on the basis of using the FYI screening tool in a community sample. Initial response rate to the FYI mailing for this intervention study was 19%. We successfully recruited 24 families (41%) of 12-month-old infants who met the FYI risk cutoffs to participate in further developmental assessments. Only 2 of these families had made contacts with community EI providers prior to receiving the FYI mailing. Recruitment of eligible participants into the RCT was 89%.

### 3.2. Utilization of Community EI Services

The second aim was to describe participating families' subsequent utilization of community EI services. All 18 infants confirmed to be at risk of developmental concerns (regardless of willingness to enroll in the RCT) at the Time 1 assessment were referred to community EI services at 13–17 months of age. Of the 16 families who enrolled in the RCT, only 2 had been previously referred to community EI services by their pediatricians. Descriptive data compiled from the monthly community EI services interviews revealed that half (8/16) of the RCT families received community EI services at some point during their participation between Time 1 and Time 3, but rates varied substantially by group. Only 4 of the 11 (36%) families in the ART treatment group received community EI services, and two of these began only after the ART intervention phase ended. Four of the 5 (80%) families in the REIM control group received community EI services, all beginning during the time of the intervention phase. The descriptive patterns were similar between groups for those children actually receiving EI services defined as (a) the average age of entry to the EI system (ART = 19.7, range = 18–22 months; REIM = 19.0, range = 16–22 months); (b) when the first EI service was provided (ART mean age = 22.0, range = 18–26, median = 21; REIM mean age = 20.5, range = 16–27, median = 21); and (c) the average number of EI services received, excluding service coordination (ART = 3.0, range 1–5; REIM = 2.5, range 1–5). The ART and REIM groups both received the same types of community services overall, which typically comprised service coordination (~1 contact per month) and 1-2 therapeutic services (i.e., speech-language therapy, occupational therapy, feeding therapy, and/or developmental/play therapy or a TEACCH play group, usually for 1 hour per week). Neither of the families in the Eligible/Declined group sought or received community EI services prior to the Time 3 diagnostic follow-up assessment.  Table 2 shows the dosage of community EI received by each group of families.

### 3.3. Findings from RCT Intervention Phase

The next aim focused on the potential of ART to improve child developmental outcomes and parental responsiveness as compared to the REIM group. Descriptive statistics for all outcome variables are in Table 3.

For each of the outcomes examined, Table 4 reports the estimates for the primary parameters of interest from the linear and multiphase growth models, as well as the AIC statistics, with lower values indicating better fit of the model to the data. Because the multiphase and linear growth models are not nested due to differences in time coding, we did not rely on likelihood ratio tests to decide which model is preferred. For the strictly linear regression model, the difference in the slope between treated and untreated subjects, *γ*, can be interpreted as the expected difference between treated and control subjects at Time 3 (or ~20 months after the first assessment). This interpretation of the parameter was obtained by dividing the time variable, *T*_*it*_, which was originally coded in months, by 20. The multiphase growth models, in contrast, contain two parameters of interest: the differential rate of growth between treatment and control (*γ*_1_) between Time 1 and Time 2 assessments (the active treatment period) and the same quantity (*γ*_2_) for the period between Time 2 and Time 3 assessments (the follow-up period).

In the linear model, significant associations between treatment assignment and posttreatment growth were observed for three outcomes: CSBS Caregiver Questionnaire Total standard score, MSEL Receptive Language *T* score, and SEQ Hyperresponsiveness mean score (see Table 4). The associations observed for the first two outcome variables were in the expected direction, with exposure to the ART intervention associated with* improved* child communication outcomes. However, parents participating in the ART intervention reported* higher* levels of SEQ Hyperresponsiveness in their children than did parents in the control condition.

**Figure 3:**
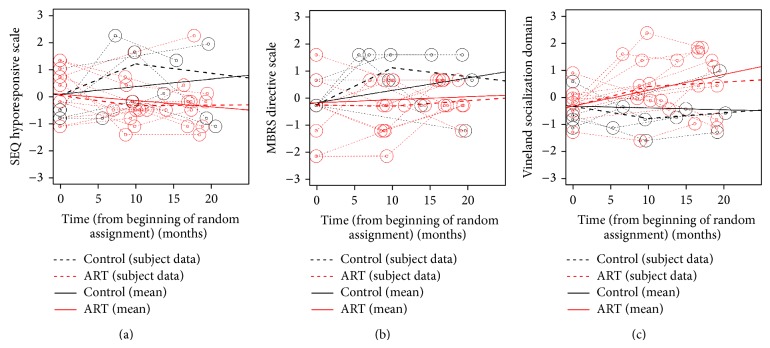
Three examples of nonlinear growth trajectories for outcomes with significant differences between ART and REIM groups: (a) SEQ hyporesponsive scale; (b) MBRS directive scale, and (c) Vineland socialization domain.

In the case of the multiphase model, statistically significant associations between treatment status and growth were concentrated in the active treatment phase (Time 1 to Time 2). Because randomization insures that parents from both experimental groups were drawn from the same baseline distributions on the outcome variables (at least in expectation), the *γ*_1_ parameter can be interpreted as the expected difference between treatment and control at the second, posttreatment measurement occasion. After the Time 1 and Time 2 assessments, 5 outcome variables were statistically significant. As expected, parents in the ART intervention group showed lower levels of MBRS Directiveness and had children who displayed better outcomes, specifically lower symptoms for SEQ Hyporesponsiveness and higher Vineland expressive and receptive communication as well as socialization. In contrast, statistically significant differential growth x treatment status in the follow-up period (Time 2 to Time 3) was largely absent. The AIC statistic indicated that the multiphase model for these 5 outcome variables provided a better fit than the linear model (Table 4); this is exemplified in the plots shown in Figures 3(a), 3(b), and 3(c).

The effect sizes for the outcome variables in Table 5 gauge the magnitude of the statistically significant effects previously revealed in Table 4. The denominator for the effect size is the estimated residual variance at the first measurement occasion. The majority of the outcomes were measured on 3 occasions, so this quantity is simply the square root of the intercept variance plus the residual variance, or $\sqrt{\left(\tau_{00}^{2}+\delta^{2}\right)}$. This particular formulation of the effect size is a regression-based analog of the familiar Cohen's D [21], which is itself a ratio of the difference in the group means and the within group standard deviations (pooled). In the current context, the reporting of effect sizes facilitates comparison of intervention impacts across outcome variables that lack a common metric.

### 3.4. Clinical Outcomes

Exploratory descriptive analyses indicated that 8 of the 18 children (44%) followed in the study obtained a diagnosis of ASD by a clinical psychologist (blind to group assignment) at Time 3. Specifically, 4/11 (36%) in the ART group (2 autistic disorder; 2 PDDNOS), 2/5 (40%) in the REIM group (both autistic disorder), and 2/2 (100%) in the Eligible/Declined group (one autistic disorder; one PDDNOS) were identified. The average scores on the MSEL across groups were within the average range, though there was considerable variability among the sample; some of the children with ASD diagnoses scored in the average to above average range on the MSEL. All of the children without an ASD diagnosis were noted by the clinical team to have other concerns (i.e., sensory processing, anxiety, articulation, fine motor, or behavior problems) at Time 3.

## 4. Discussion

This study addressed the feasibility of enrolling 12-month-old infants at risk of a later diagnosis of ASD into an RCT and tested the preliminary efficacy of ART, a parent-mediated intervention, and relative to REIM (i.e., referral to early intervention and monitoring). Utilization of community EI services and clinical diagnostic outcomes were also measured for the 18 families who met eligibility criteria for the RCT. To our knowledge, this is the first study to describe effects of a behavioral parent-mediated intervention with a community-based sample of infants at risk of ASD identified at the young age of 12 months. Overall, the findings indicated improved child receptive language, socialization, and sensory hyporesponsiveness, as well as a less directive parental interactive style for the ART group relative to the REIM group, particularly during the 6–8 month active phase of intervention.

### 4.1. Feasibility

Although response rates to the FYI screening based on mailings to families identified through the state birth registry were relatively low, they were consistent with our previous research (e.g., [41]). Some families were not reachable, but others gave reasons for declining that included lack of time or lack of concern regarding their child's development. Only 41% of families whose infants met the FYI risk score cutoffs came in for further assessment, but when the in-person assessments were concluded, 89% of those who were eligible agreed to enroll in the RCT and were subsequently randomized. This study thus demonstrated feasibility for enrolling families from a community sample into an RCT “before diagnosis,” but also stressed the challenges of ascertaining a community-based sample of infants whose parents may have limited awareness and/or acceptance of ASD-related concerns. Utilizing a two-tiered ascertainment approach (a parent report screener followed by in-person assessments) provided more opportunities for parents to witness and reflect upon their infant's behaviors in a novel context. While less than half of parents invited to participate based upon FYI results agreed to participate further, most of the eligible families enrolled in the RCT after the in-person assessment, suggesting stronger feasibility at this latter point of the process. However, not all parents who enrolled in the RCT voiced concerns about their child's development even after the assessments were completed, and, moreover, not all of those who acknowledged concerns chose to enroll. Motivations (e.g., altruism, potential health benefits) and barriers (e.g., inconvenience, mistrust) to family participation in RCTs are described in qualitative research [64–66] and cover a wide range of child, family, trial design, and provider factors. Although studies enrolling diagnosed clinical samples may have somewhat fewer challenges, the average age of enrollment tends to be higher [14, 15] and, thus, may have potential implications for prognosis and outcomes. The retention rates for families who enrolled in the present study were very high, with 100% of families completing the final assessment ~20 months after their infant was first screened. This was true of the families in the ART condition who had weekly home visits and often formed close relationships with project therapists, as well as those in the REIM (and Eligible/Declined) groups who did not have an opportunity to form those relationships, but who were contacted monthly by the project coordinator. Although other behavioral intervention studies using parent-mediated methods have shown similarly high retention rates, the majority of those studies was shorter in duration and was aimed at toddlers showing symptoms fully consistent with ASD diagnoses [11, 12, 16]. Thus, the present study expands the literature to demonstrate the feasibility for retaining a community sample of infants who are at risk of ASD “before diagnosis” in an early intervention study.

### 4.2. Subsequent Utilization of Services

Only 2 of 16 families enrolled in the RCT were referred to community EI services by their pediatricians* prior* to enrolling in the RCT; thus, 6 families (37.5%) entered the EI system sooner than would have occurred through usual primary care practices. Through participation in our early screening phase, infants deemed to be at risk of developmental concerns were referred to community EI services at 13–17 months of age. Given that the average age of diagnosis of autistic disorder in NC is 37 months [1] and that only 2 of the 18 children who we identified as at risk had been referred to EI for any type of developmental concerns prior to the FYI screening, our findings suggest that children in both the REIM and the ART groups were identified and referred earlier than standard practice for ASD screening and referral in the state of NC [1].

It is important to note that half of the families randomized to either ART or REIM were receiving community EI services by their Time 3 assessment (~30 months of age) whereas neither child in the Eligible/Declined group was receiving services by this same time point, probably because their families did not pursue the clinical team's Time 1 recommendations. Both of these children were diagnosed with ASD at their Time 3 assessments, so further research is definitely needed to determine factors influencing earlier access to services, including the extent to which monthly monitoring and support for at-risk infants (as we provided to both ART and REIM families) can facilitate such access.

An important contrast between our model and current practice is that we intervened with one-year-olds based on indicators of being* at risk *of an eventual diagnosis of ASD. In NC and many other states, documented risk of a developmental delay or disorder is not included in the eligibility criteria for publicly mandated EI services. Rather, children must either have significant current delays in one or more areas of development or must be diagnosed with an established condition. See http://www.beearly.nc.gov/index.php/providers/eligibility-referral, retrieved 8/4/2014, for a description of this policy. Thus, the findings in the present study have implications for clinical practice and government policy.

### 4.3. Promise of ART

The children who received the ART intervention outperformed the REIM group on several child developmental outcomes in the key domains of social-communication and sensory-regulatory functions. Our study was not powered to detect anything less than large effects at the .05 confidence level; thus, our significant findings are associated with large effect sizes (>.60). Such large effects were more apparent on the parent-report measures (e.g., Vineland, SEQ), but also emerged in observed measures (e.g., MSEL, MBRS). Our results add to growing empirical evidence that clearly links early pivotal behaviors (e.g., social play, joint attention, arousal and attention, engagement, adaptability, and coping, etc.) to later developmental outcomes in children with and/or without ASD [13, 33–37] and extends these findings to one-year-olds at risk of a later diagnosis of ASD who were identified via community-based screening.

Interestingly, one variable showed a pattern of change in a direction opposite to what was predicted—SEQ Hyperresponsiveness increased rather than decreased over time for the ART group. One interpretation is that as children's language and social skills increased and parents became more responsive to their child's behaviors, they may have also become more aware of their child's sensitivities and aversions and thus were more likely to report them. Future studies will be needed to explore this unexpected finding.

Although both the ART and REIM groups made gains over time, their rates and patterns of developmental change varied across variables, with some demonstrating linear growth (e.g., MSEL Receptive Language, CSBS Caregiver Questionnaire Total Score, and SEQ Hyperresponsiveness) and some demonstrating nonlinear change (i.e., SEQ Hyporesponsiveness, Vineland Receptive and Expressive Communication and Socialization, and MBRS Directive). The ART group showed the most benefit during the active treatment phase (i.e., first 6–8 months), with some attenuation in improvement in the follow-up phase. Concomitant patterns were seen with parental behaviors, such that directiveness in the ART group showed large significant improvement between Time 1 and Time 2 and then decreased in the follow-up phase. These findings underscore the importance of using multiple assessment time points (3 or more) to optimize sensitivity to potentially nonlinear treatment changes during both active and maintenance phases. Future studies with larger samples should address whether treatment fidelity and improvements in parental behaviors (e.g., responsiveness) mediate child developmental outcomes.

The present findings also suggest that studies with larger scaled trials should explore the need for booster sessions in the follow-up phase of intervention or the need to better prepared families for transitions from RCTs to conventional community EI services. Although maturational changes (i.e., “catch up” of the REIM group) could be hypothesized, these effects could not be disentangled from treatment effects in the present study. However, given that the REIM group likely benefitted from earlier identification and referral to community EI services than usual and that their boost in performance between Times 2 and 3 coincided with increases in the intensity of the EI services, we believe that maturational changes are an insufficient explanation.

### 4.4. Limitations, Future Directions, and Clinical Implications

This study adds to the growing literature indicating that earlier identification of infants at risk of ASD can lead to earlier access to EI services and can facilitate improved developmental outcomes. Although the ART group outperformed the REIM group in both child and parent outcomes, the present study has too few participants to make definitive conclusions about the efficacy of ART. However, the present study does suggest that larger scaled trials should be conducted to extend exploration of this promising intervention. Limitations of the intent-to-treat design that is commonly used in RCT such as the present study include the assumption that the treatment condition is the same as treatment exposure. Clearly, not all family decisions and variables can be controlled by randomization: families often behave differently despite being in the same condition, resulting in heterogeneity in the intent-to-treat effects.

The present study does not reveal whether a diagnosis of ASD may have been prevented through earlier behavioral intervention as theorized by some experts in the field (e.g., [26]). Approximately half of the children in the ART and REIM groups, as well as both children in the Eligible/Declined group, received an eventual diagnosis of ASD. Dawson and colleagues [13] reported that two years of intensive treatment with the Early Start Denver Model led to children having less problematic variants of ASD at around 4 years of age. This suggests that a larger-scaled study could reveal more subtle effects of ART in ameliorating ASD symptom severity or reversing early symptoms. The diagnosis of children in our sample at an average of 32 months is considerably earlier than reported in the literature for community samples (61 months) [67] and, thus, promotes earlier access to ASD specific services in the community.

Future studies should investigate the extent to which enrollment in an RCT of a behavioral intervention may postpone community EI services for some families, and whether or not this decision affects outcomes. In our study, engagement in community services differed by group. The families receiving our novel intervention were less likely to initiate community EI services, particularly during the active phase of ART. This effect may have reflected parental perceptions that their child's needs were being sufficiently addressed by our intervention. It is important to note that for both the ART and REIM groups, some infants referred for community EI services that met the state's eligibility criteria for Part C services and some did not. Moreover, some families did not seek community EI services for their infants, or they waited until an older age to do so. The community EI services received by a subset of families varied widely in goals, strategies, and intensity. Specific goals and services for children eligible for Part C services are determined through the process of developing the Individualized Family Service Plan. Some families also pursued services privately. Although researchers hope to validate beneficial effects of novel behavioral interventions, the potential costs (e.g., time, anxiety, and delay of community EI services) of entering into an RCT of an intervention that is potentially non-efficacious should not be neglected and requires further research.

Future research may also want to explore alternate strategies to identify and enroll children from community samples showing early risk signs for ASD. Working with pediatric or family medicine practices where developmental and ASD screening takes place could potentially increase family enrollment at an earlier stage. This strategy might also yield more diverse samples through targeted recruitment. Although the present study was not intended as a large-scale epidemiological study, we recognize the need to replicate findings with large, diverse samples.

To summarize, the present study confirmed the feasibility of using the FYI with a community sample to enroll 12-month-olds at risk of a later diagnosis of ASD into a behavioral RCT. The families enrolled in this study were from a narrower demographic (e.g., white/college educated) than the community at large; thus, the possibility of nonresponse bias requires further study. Although both groups made significant gains and received community services earlier than they would have through conventional surveillance and pediatric referrals, ART showed more beneficial effects relative to REIM for both child and parent outcomes, particularly in the active phase of treatment. Our study supports the promise of using a parent-mediated early intervention with very young infants at risk of ASD in authentic home contexts using an intensity of professional effort compatible with the intensity of business-as-usual EI services in our state. Future large scaled studies are needed to replicate these findings and to further assess potential mediators and moderators of comparable early behavioral interventions. Mixed methods research would be ideal to better understand quantitative outcomes as well as qualitative explanations for variability in such outcomes. Finally, scale-up and implementation efforts will need to better address the potential reasons motivating some families to seek early behavioral interventions.

## Figures and Tables

**Table 1:** Participant demographics of RCT eligible families (*n* = 18).

	ART intervention group (*n* = 11)	REIM control group (*n* = 5)	Eligible/Declined group (*n* = 2)
Chronological age in months			
Range at Time 1	13–17	13–17	17
Mean (SD)	15.22 (1.2)	15.6 (1.3)	17.7 (0.19)
Gender (boys)	9/11	5/5	2/2
Ethnicity (white)	9/11	4/5	1/2
Mother's education			
High school/vocational	1/11	—	—
College	9/11	5/5	1/2
Missing	1/11	—	1/2
MSEL early learning composite mean (SD)	86.6 (18.7)	85.8 (14.1)	81.0 (2.8)
MSEL expressive language age mean (SD)	11.9 (3.7)	10.8 (2.4)	13.5 (2.1)
MSEL receptive language age mean (SD)	13.0 (5.0)	12.8 (4.2)	14.5 (0.7)
MSEL visual reception age mean (SD)	14.9 (3.3)	16.2 (1.9)	17.5 (0.7)

Note: MSEL: Mullen Scales of Early Learning.

**Table 2:** Dosage of community EI services utilized by groups across study phases.

	ART group	REIM control group	Eligible/Declined group
Number of children receiving community EI services prior to Time 3 (NC state or private), including service coordination	*N* = 4/11 (36%)	*N* = 4/5 (80%)	*N* = 0/2 (0%)
Mean number of hours (range) of community EI services (OT, SLP, PT, feeding, or play therapy/group) between Times 1 and 2	8.78 (0–65)	29.8 (0–62)	0
Mean number of hours (range) of community EI services (OT, SLP, PT, feeding, play therapy/group, and developmental preschool) between Times 2 and 3	13.80 (0–76.3)	154.47 (0–525)	0
Mean number of hours (range) of community EI services total across study from Times 1 through 3.	22.6 (0–93.5)	184.3 (0–546)	0

**Table 3:** Means and standard deviations (SD) for all variables: ART and REIM groups.

	Time 1 assessment		Time 2 assessment		Time 3 assessment	
	Mean	SD	Mean	SD	Mean	SD
MSEL Expressive Language *T* Score						
ART	38.00	10.53	41.64	9.00	50.55	13.91
REIM	34.00	4.12	34.75	15.22	46.80	15.17
MSEL Receptive Language *T* Score						
ART	39.73	15.50	44.00	12.71	52.27	8.19
REIM	38.40	13.69	33.75	16.13	42.40	10.64
MSEL Visual Reception *T* Score^*^						
ART	46.09	12.72	51.0	9.09	54.55	10.75
REIM	50.80	12.28	48.25	10.78	47.80	18.44
MSEL Early Learning Composite						
ART	86.55	18.69	90.70	16.60	101.09	19.42
REIM	85.80	14.15	82.50	23.95	90.40	25.14
Vineland Expressive Comm. *V* Scale Score						
ART	13.91	1.70	14.82	1.99	15.55	1.968
REIM	11.80	2.04	11.25	2.36	13.20	2.58
Vineland Receptive Comm. *V* Scale Score						
ART	14.55	1.86	15.64	1.80	15.82	1.99
REIM	14.40	2.07	13.00	2.44	14.40	2.88
Vineland Communication Standard Score						
ART	94.45	8.15	99.91	8.08	102.73	9.43
REIM	86.80	12.68	82.25	14.57	92.60	10.83
Vineland Daily Living Skills Domain						
ART	91.55	9.83	101.09	13.45	99.91	12.98
REIM	86.00	5.66	82.00	13.54	95.00	7.84
Vineland Socialization Domain						
ART	93.45	7.22	101.27	13.91	104.82	14.72
REIM	89.60	8.41	84.00	6.73	91.20	10.78
Vineland Adaptive Behavior Composite						
ART	94.45	6.71	99.73	11.51	101.45	12.65
REIM	88.00	7.58	81.50	9.04	90.00	9.80
SEQ Hyperresponsiveness Scale Mean						
ART	2.01	0.34	2.10	0.43	2.31	0.40
REIM	1.93	0.47	1.91	0.19	1.80	0.24
SEQ Hyporesponsiveness Scale Mean						
ART	1.88	0.51	1.64	0.48	1.65	0.54
REIM	1.60	0.42	2.17	0.79	1.93	0.72
SPA Hyperresponsiveness Scale Mean						
ART	1.55	0.29	1.60	0.52	1.37	0.34
REIM	1.59	0.45	1.38	0.32	1.26	0.23
SPA Hyporesponsiveness Scale Mean						
ART	2.80	0.89	2.35	1.07	2.30	0.44
REIM	3.35	0.89	3.08	0.64	2.43	0.95
MBRS Directive Scale						
ART	2.90	0.61	2.95	0.52	3.18	0.34
REIM	3.20	0.27	3.88	0.25	3.40	0.65
MBRS Responsive Scale						
ART	3.08	0.75	3.70	0.77	3.97	0.57
REIM	3.38	0.35	3.30	0.24	3.46	1.12
CSBS Caregiver Questionnaire Total Standard Score						
ART	83.18	9.32	102.72	12.24	—	—
REIM	81.60	8.17	81.5	14.46	—	—
CSBS Behavior Sample Total Standard Score						
ART	87.54	15.77	97.45	15.86	—	—
REIM	81.00	11.09	83.25	21.94	—	—

^*^The Mullen Visual Reception *T* Score was not used as an outcome measure but is provided in this table to describe the sample more fully.

**Table 4:** Estimated difference in growth between treatment and control from two difference model specifications.

Outcome	Linear growth model^1^	Multiphase growth model^1^		Model fit comparison: AIC^3^	
	*γ*	*γ*_1_	*γ*_2_	Linear	Multiphase
MBRS Directive Scale Score	−0.701 (0.54)	**−1.419 (0.45)** ^*^	**1.053 (0.561)** ^+^	130.1	**123.2**
MBRS Responsive Scale Score	0.925 (0.536)	0.647 (0.541)	0.13 (0.677)	**125.5**	126.9
CSBS-Behavior Sample Total Standard Score^2^	0.896 (0.623)	NA	NA	**83.7**	NA
CSBS-Caregiver Question. Total Standard Score^2^	**1.228 (0.447)** ^*^	NA	NA	**69.7**	NA
MSEL Expressive Language *T* score	0.187 (0.716)	0.211 (0.404)	−0.252 (0.454)	116.2	**115.1**
MSEL Receptive Language *T* score	**0.928 (0.403)** ^*^	0.76 (0.485)	−0.029 (0.589)	123.4	**123.2**
SEQ: Hyperresponsive Scale Mean Score	**1.38 (0.533)** ^*^	0.452 (0.373)	0.696 (0.419)	**114.3**	117.3
SEQ: Hyporesponsive Scale Mean Score	−1.023 (0.628)	**−1.541 (0.478)** ^*^	0.738 (0.534)	131.6	**127.6**
SPA: Hyperresponsive Scale Mean Score	0.525 (0.58)	0.794 (0.519)	−0.447 (0.598)	129.0	**127.4**
SPA: Hyporesponsive Scale Mean Score	−0.31 (0.517)	−0.701 (0.53)	0.555 (0.679)	131.0	**130.6**
Vineland Expressive Communication *V*-scale score	0.614 (0.575)	**0.79 (0.328)** ^*^	−0.474 (0.356)	108.8	**106.9**
Vineland Receptive Communication *V*-scale score	0.874 (0.594)	**1.34 (0.356)** ^*^	**−0.765 (0.393)** ^+^	120.4	**113.0**
Vineland Socialization Standard Score	**1.299 (0.734)** ^+^	**1.178 (0.431)** ^*^	−0.092 (0.51)	117.1	**115.3**

^1^Standard errors in parentheses; ^2^outcome was only measured on two occasions; ^3^ **lower** AIC statistics indicate better fit between linear and multiphase models (indicated in bold font between last two columns).

^+^ **P** < 0.10; ^*^ **P** < 0.05.

**Table 5:** Effect sizes from linear and multiphase growth models with statistically significant outcomes.

	Effect size^2^		
	Linear	Multiphase	
	Difference at Time 3	Difference at Time 2	Difference at Time 3
MBRS Directive Scale Score	−0.642	**−1.379**	**−0.356**
CSBS Caregiver Questionnaire Total Standard Score^1^	**2.022**	NA	NA
MSEL Receptive Language *T* Score^1^	0.876	**0.704**	0.678
SEQ Hyperresponsiveness Scale Mean Score^1^	1.441	**0.470**	1.194
SEQ Hyporesponsiveness Scale Mean Score	−1.187	**−1.684**	−0.877
Vineland Expressive Communication *V*-scale Score	0.701	**0.940**	0.376
Vineland Receptive Communication *V*-scale Score	0.972	**1.514**	**0.650**
Vineland Socialization Standard Score	1.968	**1.852**	1.708

^1^Linear model provided better fit than multi-phase.

^2^Effect sizes: low (<0.30), medium (0.30–0.60), and high >0.60 (Cohen, 1988 [21]).

Bold font indicates effects that were statistically significant.
